# The Multimorbidity Knowledge Domain: A Bibliometric Analysis of Web of Science Literature from 2004 to 2024

**DOI:** 10.3390/healthcare13212687

**Published:** 2025-10-23

**Authors:** Xiao Zheng, Lingli Yang, Xinyi Zhang, Chengyu Chen, Ting Zheng, Yuyang Li, Xiyan Li, Yanan Wang, Lijun Ma, Chichen Zhang

**Affiliations:** 1 School of Public Health and Management, Guangzhou University of Chinese Medicine, Guangzhou 510006, China; zxiao125@sina.com; 2 Key Laboratory of Philosophy and Social Sciences of Guangdong Higher Education Institutions for Collaborative Innovation of Health Management Policy and Precision Health Service, Guangzhou 510515, China; lilyyang0819@163.com (L.Y.); chenchengyyu@163.com (C.C.); zhti2002@163.com (T.Z.); liyuyang118@163.com (Y.L.); 18894136919@163.com (X.L.); 15310058873@163.com (Y.W.); 3 School of Health Management, Southern Medical University, Guangzhou 510515, China

**Keywords:** multimorbidity, health services, bibliometric analysis, knowledge graph analysis

## Abstract

**Aim:** With the intensification of population aging, the public health challenges posed by multimorbidity have become increasingly severe. This study employs bibliometric analysis to elucidate research hotspots and trends in the field of multimorbidity against the backdrop of global aging. The immediate aim is to systematically map the intellectual landscape and evolving patterns in multimorbidity research. The ultimate long-term aim is to provide a scientific basis for optimizing chronic disease prevention systems and guiding future research directions. **Methods:** The study adopted the descriptive research method and employed a bibliometric approach, analyzing 8129 publications related to multimorbidity from the Web of Science Core Collection. Using CiteSpace, we constructed and visualized several knowledge structures, including collaboration networks, keyword co-occurrence networks, burst detection maps, and co-citation networks within the multimorbidity research domain. **Results:** The analysis included 8129 articles from 2004 to 2024, published across 1042 journals, with contributions from 740 countries/regions, 33,931 institutions, and 40,788 authors. The five most frequently occurring keywords were prevalence, health, older adult, mortality, and risk. The top five contributing countries globally were the United States, the United Kingdom, Germany, China, and Spain. Five pivotal research trajectories delineate the intellectual architecture of this field: ① Evolution of Disease Cluster Management: Initial investigations (2013–2014) prioritized disease cluster coordination within general practice settings, particularly cardiovascular comorbidity management through primary care protocols and self-management strategies. ② Paradigm Shifts in Health Impact Assessment: Multimorbidity outcome research demonstrated sequential transitions—from physical disability evaluation (2013) to mental health consequences like depression (2016), culminating in current emphasis on holistic health indicators including frailty syndromes (2015–2019). ③ Expansion of Risk Factor Exploration: Analytical frameworks evolved from singular physical activity metrics (2014) toward comprehensive lifestyle-related determinants encompassing behavioral and environmental dimensions (2021). ④ Emergence of Polypharmacy Scholarship: Medication optimization studies emerged as a distinct research stream since 2016, addressing therapeutic complexities in multimorbidity management. ⑤ Frontier Investigations: Cutting-edge directions (2019–2021) feature cardiometabolic multimorbidity patterns and their dementia correlations, signaling novel interdisciplinary interfaces. **Conclusions:** The prevalence of multimorbidity is on the rise globally, particularly in older populations. Therefore, it is essential to prioritize the prevention of cardiometabolic conditions in older adults and to provide them with appropriate and effective health services, including disease risk monitoring and community-based chronic disease care.

## 1. Background

With the accelerated global aging population and the growing burden of chronic diseases, multimorbidity, the co-occurrence of two or more chronic conditions in an individual, has become a major challenge in public health and clinical medicine [1]. According to the World Health Organization (WHO), chronic diseases account for over 70% of global mortality, and the prevalence of multimorbidity exceeds 50% among older adults, with a rapid increase observed in low- and middle-income countries [2]. Compared to single-disease conditions, multimorbidity significantly increases health risks, healthcare costs, and societal caregiving burdens, while complicating clinical decision-making and therapeutic outcomes [3]. This issue has led to a shift in academic focus, moving away from traditional single-disease-centered approaches to investigating the pathological mechanisms, management strategies, and health outcomes of coexisting conditions. Particularly following the emergence of COVID-19, evidence has indicated that multimorbidity, especially cardio-metabolic multimorbidity and polypharmacy, is associated with an elevated risk of COVID-19 infection [4]. Moreover, multimorbidity has been established as an independent risk factor for mortality in patients hospitalized with COVID-19 [5].

Recent developments in multimorbidity research reflect an integration of multiple disciplines. Epidemiological studies using large-scale cohort data have revealed prevalence patterns and social determinants of multimorbidity [6]. Clinical research has concentrated on polypharmacy interactions and personalized interventions [7,8], while health management fields emphasize the creation of integrated care models [9]. Despite progress, significant challenges remain: The diversity of disease combinations complicates the establishment of standardized definitions and classification systems [10]. The dynamic interactions among biological, psychological, and social factors are still not fully understood [11], and evidence-based guidelines for coordinated multimorbidity management remain limited [11]. In light of these issues, systematically mapping research hotspots and emerging trends in this field is crucial for advancing both theoretical knowledge and practical implementation.

However, despite the proliferation of studies, the knowledge structure, evolutionary pathways, and emerging trends in multimorbidity research have not been systematically examined with bibliometric rigor. Existing reviews often focus narrowly on clinical or epidemiological aspects, lacking a holistic mapping of the field’s intellectual landscape and collaborative networks. For instance, Xu [12] delineated the global research architecture up to 2016, identifying significant disparities in research output between high-income and low- and middle-income countries, as well as a misalignment between disease burden and scientific focus. However, their analysis did not encompass recent advancements, particularly in the realm of interdisciplinary integration and emerging thematic clusters. Furthermore, Zhou [13] recently conducted a bibliometric study concentrating on mental health and multimorbidity among older adults, which highlighted depression and anxiety as central issues. Nevertheless, their investigation was confined to the mental health dimension and failed to offer a holistic perspective of the field’s overall intellectual structure and evolutionary pathways.

There is a lack of comprehensive, systematic, and up-to-date bibliometric analysis that can fully capture the knowledge structure, collaborative networks, and development trends within the field of multimorbidity research. Unlike previous reviews, which often focused on specific regions, themes, or disciplinary domains, this study employs an integrated approach combining bibliometric and knowledge mapping methods to systematically examine the evolutionary trajectory, core knowledge clusters, and methodological advances in multimorbidity research. Using data from the Web of Science Core Collection and utilizing CiteSpace software (v 6.3 R1), the direct aim of this is to delineate the disciplinary development pathways, identify key technological and conceptual progress, and highlight understudied areas. The ultimate aim of this study is to provide a scientific basis for formulating chronic disease prevention and control policies, updating clinical practice guidelines, and guiding future research directions.

## 2. Methods

### 2.1. Data Sources

(1) Literature Search Strategy

The data for this study were retrieved from the Web of Science Core Collection Citation Index. A comprehensive search strategy was employed using the query: Topic = (“multimorbidity”) OR Topic = (“multiple chronic condition”) OR Topic = (“multiple chronic disease*”), with no start date restriction and an end date of 11 December 2024. The search was initially limited to the document type “Article”.

(2) Literature Screening Criteria

Inclusion criteria included articles that were selected based on those retrieved through the formal literature search strategy. The language was set to English. Exclusion criteria included publications with incomplete content, unclear thematic focus, and non-article document types. Initial screening identified 8141 records that met the basic criteria. To ensure the scientific integrity of the data, a manual item-by-item verification of all downloaded records was conducted in strict accordance with the predefined inclusion and exclusion criteria. Following this rigorous manual review, 8129 valid records were retained for final analysis. Bibliographic metadata, including authorship, article titles, abstracts, and citation networks, were systematically extracted. The initial year of the data is 2004. All data were retrieved and archived on 11 December 2024.

### 2.2. Methodology

(1) CiteSpace software

Bibliometrics is the study of academic publications, employing statistical methods to analyze published information and its associated metadata (e.g., abstracts, keywords, citations). It aims to describe and visualize relationships between published works and uncover hidden patterns within a body of literature. This study is a bibliometric analysis designed to investigate the published literature in the field of multimorbidity. The annual publication counts of multimorbidity-related articles from the Web of Science Core Collection were analyzed to evaluate global research trends through frequency statistics. CiteSpace software [14] was utilized to visualize the dynamic evolution and frontier trends in multimorbidity research by constructing keyword co-occurrence networks, document co-citation networks, and country/institutional collaboration networks.

**Figure 1:**
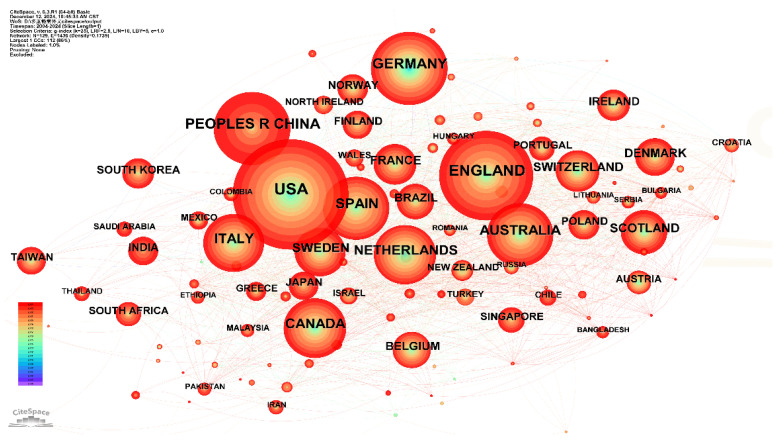
Country Collaboration Network of Multimorbidity Research Indexed in WOS.

(2) Parameter settings
  a. Following data deduplication and screening, the literature dataset (2004–2024) was segmented into 21 annual intervals.
  b. Within the CiteSpace framework, the g-index functions as a primary selection criterion for determining node inclusion or exclusion within each time slice via an adapted g-index weighting mechanism. The proportional factor k is adjustable to control the density of nodes displayed in the knowledge map. Specifically, higher values of k increase the number of nodes visualized, while lower values reduce their representation. The current parameter settings are clearly documented in the upper-left corner of each generated visualization.
  c. Separate analyses were conducted for keywords, references, and countries/institutions. In keyword co-occurrence networks (Figure 1), node size corresponds to keyword frequency, while color gradients and ring thickness reflect temporal distribution and annual publication volume, respectively (see color legend, lower left). Connecting lines denote co-occurrence relationships, with line thickness indicating frequency and color representing the first co-occurrence year [15].
  d. Burst detection algorithms identified prominent keywords marked by purple outer rings, and nodes with betweenness centrality > 0.1 were defined as pivotal hubs bridging research domains [16]. Burst detection algorithms identified prominent keywords marked by purple outer rings, and nodes with betweenness centrality > 0.1 were defined as pivotal hubs bridging the research topic.
  e. For recent trend analysis (2015–2024), co-citation clustering was performed using 10 annual slices, log-likelihood ratio (LLR, *p* < 0.001) for cluster labeling, and modularity (Q > 0.3) and silhouette (S > 0.7) metrics to validate cluster robustness.

## 3. Results

### 3.1. Spatiotemporal Distribution of Multimorbidity Research

#### 3.1.1. Temporal Distribution of Multimorbidity Research

The analysis included 8129 articles published across 1042 journals, with contributions from 740 countries/regions, 33,931 institutions, and 40,788 authors. Over time, global publication output on multimorbidity has consistently increased (Supplementary Materials Figure S1). Annual publications exceeded 100 articles in 2012 (116 articles), reaching 976 articles by 2021 (data retrieval date: 11 December 2024).

#### 3.1.2. Spatial Distribution of Multimorbidity Research

The country collaboration network of multimorbidity research indexed in Web of Science (WOS) is illustrated in Figure 1, comprising 129 nodes and 1436 connections (Density = 0.1736). The map highlights 50 countries with publication outputs exceeding 25 articles. The top five contributing countries globally were: the United States (1931 articles), the United Kingdom (1311 articles), Germany (889 articles), China (859 articles), and Spain (679 articles). The network displayed dense interconnections, with red-hued links representing increased international collaborations in this field in recent years.

### 3.2. Research Hotspots and Evolution of Multimorbidity Research

**Figure 2:**
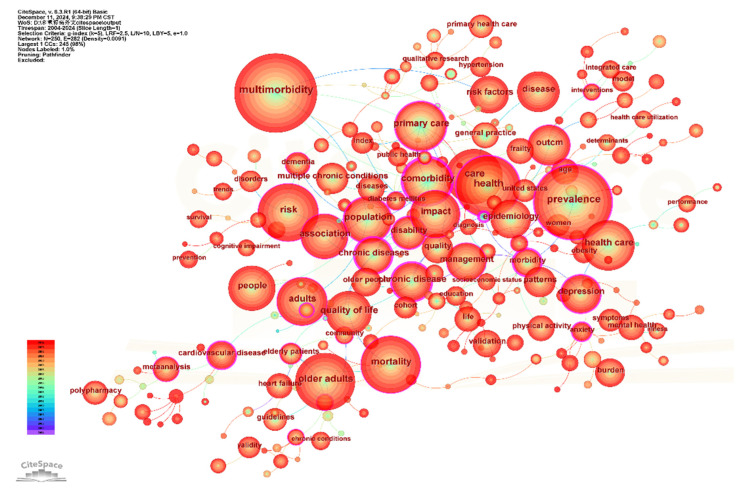
Keyword Co-occurrence Network of Multimorbidity Research Indexed in WOS.

The keyword co-occurrence network, derived from multimorbidity-related literature indexed in WOS and consisting of 250 nodes with 282 interconnections, is shown in Figure 2. The visualization highlights keywords appearing more than 100 times. After excluding search terms, the five most frequently occurring keywords were: prevalence (1713), health (1171), older adult (1047), mortality (1035), and risk (1008). Centrality analysis identified seven keywords with high betweenness centrality, suggesting key positions within the network: family practice (0.64), morbidity (0.57), depression (0.32), health (0.31), disability (0.31), prevalence (0.26), and cardiovascular disease (0.26). These keywords serve as conceptual anchors, connecting different clusters and reflecting dense co-occurrence patterns that link major themes in the field. Together, the frequency and centrality metrics outline the conceptual structure and knowledge flow in multimorbidity research. In summary, the research hotspots include older adults, mental comorbidities, cardiovascular diseases, and prevalence.

Drawing from co-occurrence networks, frequency distributions, and the timing of keyword appearances, global research hotspots in multimorbidity were organized as presented in Supplementary Materials Table S1. Five thematic clusters were identified: #1 Epidemiology, #2 Risk Factors, #3 Health Services Research, #4 Disease Patterns, and #5 Medication Appropriateness. Temporal analysis revealed evolving thematic emphases: In the early phase, studies concentrated on broad prevalence patterns (prevalence, 2004) and epidemiological characterizations (epidemiology, 2004), alongside general explorations of health outcomes (health, 2004). In the middle phase, research moved toward more detailed evaluations of health impact, with increasing attention to mental health comorbidities (depression, 2006; disability, 2007; anxiety, 2016), and identification of contributing risk factors (risk, 2009). In the recent phase, emphasis has shifted to socio-environmental factors (socioeconomic status, 2014; social determinants, 2022), environmental risks (air pollution, 2023), and behavioral contributors (lifestyle, 2024). Health service-related topics have been a constant thread, including family practice (2005), interventions (2012), and long-term care (2024). Multimorbidity patterns (2021) continue to pose complex challenges, often examined through cluster analysis (2018). A current focus is cardiometabolic multimorbidity (2022), now recognized as a central disease subtype under investigation.

### 3.3. Frontiers and Development in Multimorbidity Research

#### 3.3.1. Burst Terms in Multimorbidity Research Indexed by WOS

**Figure 3:**
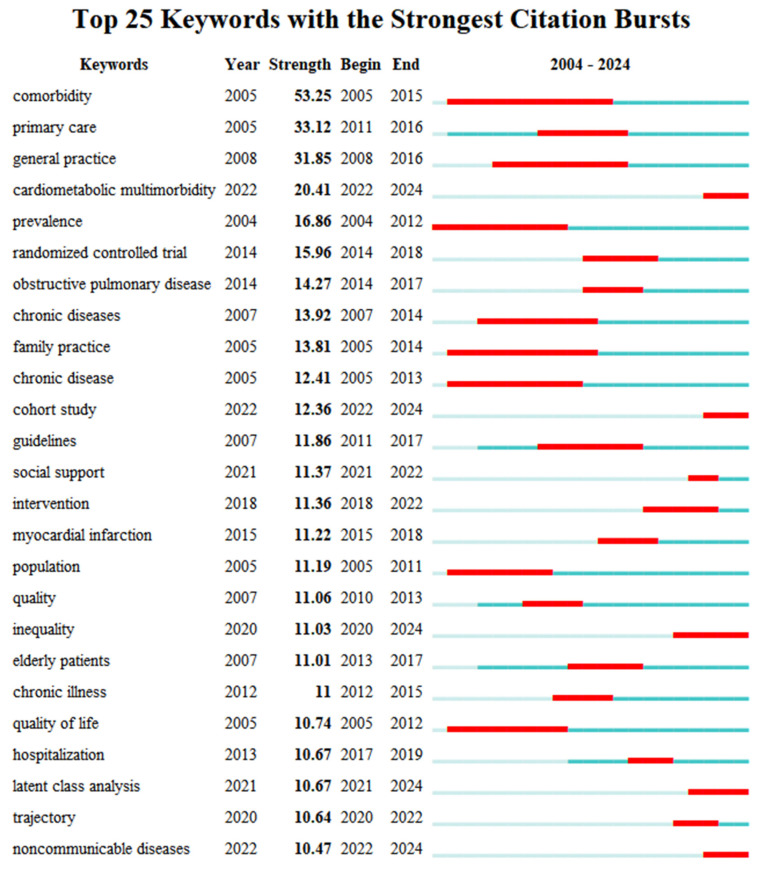
Burst Keywords in Multimorbidity Research Indexed in WOS.

Emerging directions in multimorbidity research can be identified by tracking sharp increases or notable shifts in keyword frequencies across different time periods. These terms, referred to as “burst terms”, are determined based on the rate of frequency growth in titles, abstracts, and keywords. The burst strength quantifies the intensity of focus on a given topic [17]. Using the burst detection function in CiteSpace software, prominent burst terms were extracted (Figure 3). Excluding the search term itself, “primary care” and “general practice” recorded the highest values, highlighting the field’s strong focus on disease care. From a demographic perspective, “elderly patient” (11.01) highlighted the ongoing emphasis on older adults within multimorbidity research. Analysis of recent burst terms revealed rising interest in “cardiometabolic multimorbidity” (20.41), suggesting this may become a key direction moving forward. The emergence of “inequality” (11.03) pointed to an increasing focus on social determinants, indicating that health inequality is gaining prominence as a research frontier. In addition, “cohort study” (12.36) and “latent class analysis” (10.67) indicated the frequent adoption of these study designs and analytical methods in recent investigations.

#### 3.3.2. Co-Citation Analysis of Multimorbidity Research Indexed in WOS

(1) Co-citation Analysis of Multimorbidity Literature (2004–2024)

**Figure 4:**
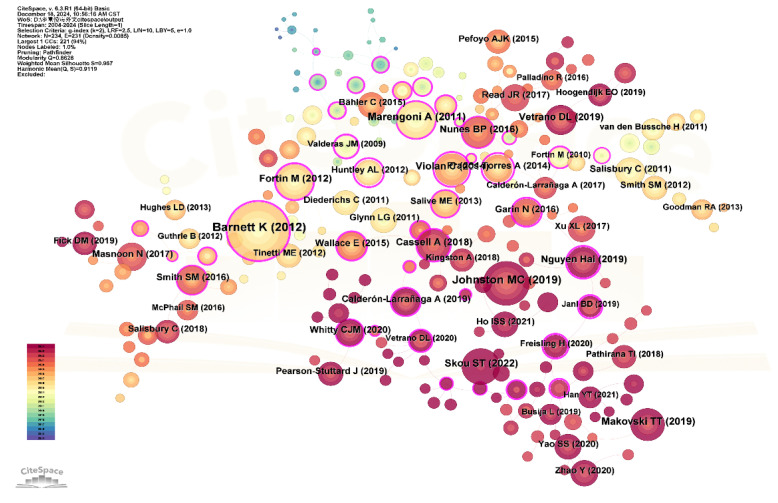
Co-citation Network of Multimorbidity Research Indexed in WOS.

A co-citation relationship occurs when two or more publications are cited together by later studies [14]. To identify research frontiers and trends within multimorbidity, we conducted a co-citation analysis of literature published between 2004 and 2024. Figure 4 presents the co-citation network from WOS-indexed literature, comprising 299 nodes and 296 links, representing references cited at least 55 times.

The top 10 most frequently co-cited articles, identified through computational analysis, were listed in Supplementary Materials Table S2. These articles form two dominant temporal clusters: 2011–2014 and 2018–2019. Of the 11 most-cited works, 8 were systematic reviews. Most focused on prevalence studies (10 articles) and disease pattern analysis, while three addressed connections with quality of life, frailty, and mortality. One study was dedicated to geriatric multimorbidity. The most frequently co-cited article assessed the effects of multimorbidity on healthcare services. Among these, the influential study by Barnett et al. [18] recorded the strongest citation burst, reflecting lasting academic interest and potential as a directional marker for future investigations.

(2) Co-Citation Clustering Analysis of Multimorbidity Research (2015–2024)

**Figure 5:**
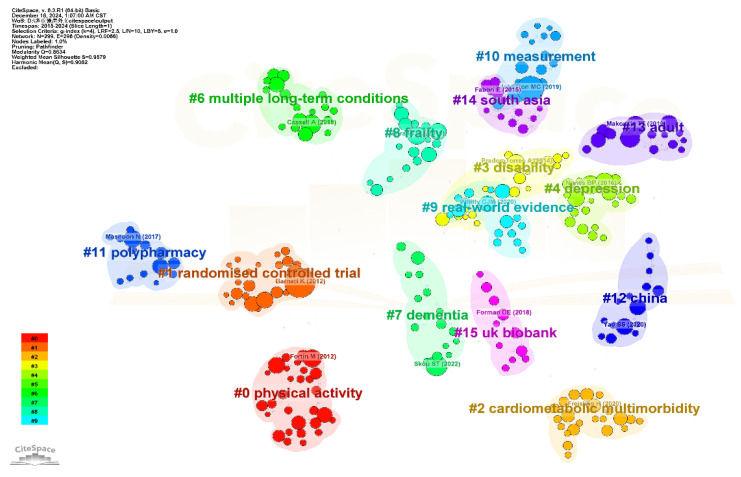
Co-Citation Clustering Network of Multimorbidity Research Indexed by WOS.

To identify recent trends and thematic directions over the past decade, we carried out a co-citation analysis of multimorbidity publications from 2015 to 2024. Clustering was performed using keywords and the LLR algorithm, producing 16 clusters (Figure 5). The modularity score (Q = 0.86) and silhouette score (S = 0.96) indicated strong structural consistency in the clustering. Detailed characteristics of each cluster are summarized in Supplementary Materials Table S3.

Five principal thematic developments were identified, with representative publications from each cluster shown in Figure 5: Focus on Disease Clusters. Early research focused on disease clusters, particularly within general practice, emphasizing management of cardiovascular diseases and associated conditions, including approaches in primary care and self-management. This trend appeared in clusters #1 and #5 (2013–2014) and later expanded to the management of multiple chronic diseases, as reflected in clusters #10 and #6. Shift in Health Impact Research. Studies examining the health impacts of multimorbidity have progressed over time, initially centering on physical health outcomes such as disability (2013), then shifting toward mental health concerns like depression (2016), and more recently emphasizing overall health, including frailty (2015, 2016, 2019). These thematic changes are captured in clusters #3, #4, #14, #8, and #9. Research on Risk Factors. Exploration of factors associated with multimorbidity began with physical activity (2014) and gradually expanded to include a wider range of lifestyle-related factors by 2021. These developments are mainly reflected in clusters #1 and #7. Unique Direction in Medication Research. Cluster #11 marks a distinct research stream focusing on medication-related issues in the context of multimorbidity, with a noticeable increase in attention beginning in 2016. Emerging Trends. Clusters #2, #13, and #15 are linked to cardiometabolic multimorbidity, while cluster #7 highlights research on dementia. These clusters, dated from 2019 to 2021, point to newly emerging areas of interest within the field.

Burst detection results identified four emerging frontier publications (Supplementary Materials Figure S2). These included a study by Cassell A on primary care (burst strength: 32.28, 2020), research by Johnston MC (29.66, 2021) and Ho ISS (25.90, 2022) on multimorbidity measurement methods, and a study by Yang Zhao on health services and health inequality related to multimorbidity (28.14, 2022).

## 4. Discussion

The analysis included 8129 articles from 2004 to 2024, published across 1042 journals, with contributions from 740 countries/regions, 33,931 institutions, and 40,788 authors. Five pivotal research trajectories delineate the intellectual architecture of this field: ① Evolution of Disease Cluster Management, ② Paradigm Shifts in Health Impact Assessment, ③ Expansion of Risk Factor Exploration, ④ Emergence of Polypharmacy Scholarship, and ⑤ Frontier Investigations. These five trajectories provide a comprehensive framework for understanding the field. Below is the detailed analysis. Below is the detailed analysis.

### 4.1. Developmental Trends in Multimorbidity Research

Globally, publication output on multimorbidity has followed a steep upward trajectory since 2004, mirroring the rapid evolution of the field. As a pressing issue in the context of chronic disease progression, multimorbidity has drawn increasing attention from researchers across disciplines. The conceptual origins of multimorbidity are rooted in the earlier notion of comorbidity, introduced by Feinstein [19]. Over time, both “comorbidity” and “multimorbidity” have gained broad academic acceptance [20]. In 2008, the World Health Organization provided a formal definition of multimorbidity as “the coexistence of two or more chronic conditions in an individual” [21], laying a foundational framework that has shaped the direction of research since.

### 4.2. Prevalence of Multimorbidity

A search of the Web of Science using the keywords “prevalence” and “multimorbidity” identified 46 highly cited publications published between 2011 and 2020, with the top 10 most cited articles concentrated between 2011 and 2015. The most cited among them was a cross-sectional study by Barnett et al. [18]. The study reported that 23.2% of patients had multiple chronic conditions. It also confirmed that multimorbidity becomes significantly more common with age, particularly among individuals aged 65 and above. A foundational review on aging and multimorbidity by Marengoni et al. [22], cited 1260 times, systematically examined the relationship between aging and multimorbidity, along with its severe impacts. It reported that multimorbidity prevalence among older adults ranges from 55% to 98%. Cross-sectional and longitudinal studies consistently showed that older age, female gender, and low socioeconomic status are major associated factors. Key consequences include disability, functional decline, reduced quality of life, and high healthcare costs. More recently, a widely cited study by Kingston et al. [23], with 164 citations, projected a continued rise in multimorbidity prevalence over the next two decades. Global epidemiological evidence now confirms that multimorbidity is a defining feature of chronic disease today. As age increases, the likelihood of having multiple chronic conditions also rises, positioning older adults as the most affected group.

In summary, available research suggests that the global prevalence of multimorbidity in older adults falls between 55% and 98%, with projections pointing to further increases in the coming 20 years. Variations across studies are mainly due to differences in geographic, demographic, and methodological factors. Despite this, the overall body of evidence clearly shows that multimorbidity is widespread in older populations. Given this, it is critical to focus on preventing complex multimorbidity in older adults and ensuring they receive appropriate, effective health services. Furthermore, future studies should emphasize clear research design—including sample sources and characteristics—and employ multiple analytical methods for cross-validation to enhance data reliability.

### 4.3. Research on Multimorbidity Patterns

Research into multimorbidity patterns represents a key focus in this field. A search on the Web of Science using the keywords “multimorbidity” and “patterns” identified several highly cited studies, including a review by Violan et al. [24]. This review analyzed 39 studies involving 70,057,611 patients from 12 countries and found that commonly observed patterns included osteoarthritis in combination with cardiovascular or metabolic conditions. Another widely cited review by Prados-Torres [10], with 279 citations, examined 14 studies and identified three recurring multimorbidity patterns: (1) cardiovascular and metabolic conditions, (2) psychological conditions, and (3) musculoskeletal diseases. In 2016, Prados-Torres also conducted a cross-sectional study across several countries, revealing that hypertension, cataracts, and arthritis were the most frequently co-occurring conditions, with each country displaying two to three dominant multimorbidity patterns. These were categorized as “cardio-pulmonary” (angina, asthma, and chronic obstructive pulmonary disease), “metabolic” (diabetes, obesity, and hypertension), and “psychiatric-musculoskeletal” (arthritis and depression). Additional influential work has come from research teams led by scholars such as Marengoni et al. [25] and van den Bussche et al. [26], focusing specifically on patterns of multimorbidity in older adults. These studies are widely cited within the field. In recent years, however, standalone research on disease patterns has declined, with growing emphasis on integrating pattern analysis with studies on etiology and contributing factors, as demonstrated by the work of Puri & Singh [27] and Lee et al. [28].

In summary, research on multimorbidity patterns continues to hold significant importance, with the three primary patterns identified as: (1) cardiometabolic conditions, (2) mental health disorders, and (3) musculoskeletal disorders. Nevertheless, the field still faces major limitations, particularly the lack of standardized and comprehensive criteria for selecting chronic diseases. This leads to considerable variation in the number and types of conditions included across different studies, thereby increasing the risk of identifying spurious associations in pattern recognition. To advance the field, it is imperative to establish validated inclusion criteria for chronic diseases based on research objectives and operational feasibility. When analyzing multimorbidity patterns, special caution is required to distinguish whether observed disease clusters reflect true associations or random co-occurrence.

Future studies should employ robust statistical models to quantify the strength of disease associations, integrate multi-omics approaches to explore underlying mechanisms, and prioritize causal inference through longitudinal cohort designs. Such efforts will facilitate the translation of research findings into clinical prevention and precision intervention practices. Furthermore, the prevention and management of cardiometabolic multimorbidity should be prioritized as a key direction for future research.

### 4.4. Research on Risk Factors of Multimorbidity

Research on the risk factors contributing to multimorbidity has emerged as a key direction in the field, with scholars globally adopting a range of methodological approaches to explore this topic. Violan et al. [24], in a systematic review, identified age, lower socioeconomic status, and sex as central factors associated with multimorbidity. Alongside these demographic indicators, other studies have shown strong links between health behaviors and the onset of multimorbidity. For example, Canizares et al. [29], in a longitudinal study, reported a higher prevalence of multimorbidity among individuals characterized by female sex, lower income, obesity, smoking, and sedentary lifestyles, identifying obesity and low income as major contributors. Kivimaki et al. [30] demonstrated that increasing BMI raises the risk of cardiometabolic multimorbidity, positioning overweight and obesity as key risk factors. Similarly, Loprinzi [31] developed a multimorbidity index to examine the association between sedentary behavior and the burden of multimorbidity, confirming a strong connection. Nicholson [32], drawing on Canadian longitudinal data, identified sleep disorders as additional potential risk factors, with notable variations by age and sex. Some studies have also explored the relationship between health behaviors and life expectancy in people with multimorbidity. For instance, Chudasama et al. [33] found that adopting healthy lifestyle behaviors could extend life expectancy by 6.3 years in males and 7.6 years in females living with multimorbidity. Psychological variables have been increasingly recognized as well, with longitudinal studies pointing to associations between mental health conditions and the development of multimorbidity [34].

Recognizing and addressing risk factors early can substantially reduce the likelihood of multimorbidity in older populations. Research from both domestic and international teams confirms that demographic characteristics such as gender and socioeconomic status [35,36] play a significant role in the onset and progression of multimorbidity. Health behavior factors, particularly smoking, sedentary lifestyle, and obesity, have consistently emerged as key drivers. Over time, research in this area has expanded, with longitudinal cohort studies providing increasingly detailed insights.

Despite this progress, a comprehensive risk assessment model for multimorbidity has yet to be established. Developing such models should be a priority for future research. Translating known risk factors into practical tools would enable public health professionals to better identify high-risk groups and implement targeted, timely interventions.

### 4.5. Health Consequences and Healthcare Burden of Multimorbidity

Analysis of research hotspots in multimorbidity highlights that the health consequences of multimorbidity are a central focus and continue to develop. Evidence consistently links multimorbidity to higher mortality rates, poorer quality of life, physical functional decline, and mental health issues. Early identification and intervention can significantly improve the quality of life and extend the healthy lifespan of older adults. A classic international study by Read [34], which conducted a meta-analysis of 40 studies involving 381,527 individuals, confirmed the association between multimorbidity and depression. Vetrano [37] provided a systematic review on the link between frailty and multimorbidity, finding a correlation between the two, though noting that further cohort studies are needed to clarify their causal relationship and developmental trends. Nunes [38] conducted a systematic review and meta-analysis of 26 studies, confirming that multimorbidity increases patient mortality risk.

In addition to its health consequences, multimorbidity presents significant disease burdens and challenges for public health systems. Cross-sectional and cohort studies by Bahler [39], McPhail [40], and Lehnert [41] showed that multimorbidity imposes substantial healthcare burdens. Salisbury [42] highlighted that individuals with multimorbidity have higher consultation rates but receive less continuous care compared to those without multiple chronic conditions. This finding emphasizes the growing demand for outpatient services and suggests that care for multimorbidity is limited, with many individuals not receiving the coordinated treatment they require. The increasing prevalence of multimorbidity presents mounting challenges for global health systems.

In summary, multimorbidity represents a significant burden on global health services, particularly among older populations. As the population ages and the prevalence of multimorbidity rises, the healthcare burden in China is also growing. China’s healthcare reforms should focus on integrated care models and patient-centered services. During outpatient care, the co-occurrence of multiple chronic conditions should be addressed with timely diagnosis and tailored treatment plans. Moreover, primary healthcare should aim to reduce the prevalence of multimorbidity and its negative effects in older adults, thereby improving health outcomes and extending life expectancy.

### 4.6. Research on Healthcare Services for Multimorbidity

Current international research on healthcare services for multimorbidity predominantly focuses on therapeutic interventions for patients with multiple chronic conditions, emphasizing diagnosis, treatment, and care, particularly in managing polypharmacy. A key contribution in this area is the multimorbidity care model proposed by Palmer et al. [43], developed through expert consensus within the European Joint Action on Chronic Diseases and Promoting Healthy Ageing (JA-CHRODIS). This model encompasses five domains and 16 actionable measures, including Delivery System Design, Decision Support, Self-Management Support, Clinical Information Systems, and Community Resource Integration. The model aims to establish a flexible framework for patient-centered care, independent of specific disease combinations, prioritizing service delivery over systemic restructuring (e.g., governance and financing). Boyd [44] introduced a multimorbidity action framework designed to help clinicians tailor care plans to individual patient needs. Tang [45] further identified seven critical intervention elements and 23 behavior-change strategies for medication management in multimorbid populations in Singapore, developed through a two-round Delphi consensus process.

Numerous studies have investigated interventions to improve health outcomes for patients with multimorbidity. For example, Smith et al. [46] conducted a meta-analysis of 17 intervention studies to inform policies for managing multimorbidity in primary care and community settings. The analysis found that in 11 randomized controlled trials, primary interventions focused on reorganizing multimorbidity care delivery, typically through case management or enhanced multidisciplinary teamwork. The remaining six studies involved patient-centered interventions, such as providing direct education or supporting self-management. Sinnott [47] developed the Behavior Change Wheel, a novel framework for intervention design, to identify strategies for medication management in multimorbidity patients. Additionally, Pascual [48] performed a literature review of five studies analyzing the effects of telemedicine interventions on mortality, hospital admissions, emergency department visits, and health-related quality of life in patients with multimorbidity.

However, the review did not find definitive evidence supporting the effectiveness of telemedicine interventions. Instead, future strategies should prioritize the development of interventions centered on self-management and aimed at behavior change, which represent a critical direction for addressing multimorbidity.

## 5. Limitations

A key strength of this study is its use of bibliometric and knowledge graph analyses to uncover large-scale research trends, collaborative networks, and thematic evolution in multimorbidity research, insights often beyond the reach of conventional systematic reviews. However, several limitations should be noted. To ensure the inclusion of high-quality publications, we selected only the Web of Science database as the data source. However, this approach may lead to the omission of some studies published in non-core journals, which could potentially represent significant research findings. Furthermore, we have not conducted a detailed analysis of research topics such as the prevalence and patterns of multimorbidity across different countries or income groups. To advance the research agenda, we recommend conducting topic-specific systematic reviews that incorporate a wider range of databases, performing secondary analyses of data at a global level, and initiating primary longitudinal cohort studies.

## 6. Conclusions

In summary, multimorbidity is increasingly prevalent worldwide, especially among older adults, and is associated with more severe psychological issues, greater physical functional impairment, and increased economic burdens. Cardiometabolic multimorbidity, in particular, represents both a common clinical pattern and an emerging research priority. Moving forward, healthcare systems should prioritize the prevention and management of cardiometabolic conditions in aging populations and ensure the provision of appropriate and effective health services. The findings of this study offer valuable insights for shaping global health policies and service planning, highlighting the need for integrated care models and evidence-based strategies to address the growing challenge of multimorbidity.

## Data Availability

The raw data supporting the conclusions of this article will be made available by the authors upon request since the data used in this study are publicly accessible from Web of Science using our search strategy.

## Supplementary Materials

The following supporting information can be downloaded at: https://www.mdpi.com/article/10.3390/healthcare13212687/s1, Figure S1: Annual Publication Volume in Multimorbidity Research; Figure S2; Prominent Publications in Multimorbidity Research Indexed by WOS; Table S1: Keyword Frequency in Multimorbidity Research Indexed in WOS; Table S2: Highly Cited Articles in Multimorbidity Research Indexed in WOS. Table S3: Clustering Information of Highly Cited Publications in Multimorbidity Research Indexed by WOS.
