# Supplementary material for: The Multimorbidity Knowledge Domain: A Bibliometric Analysis of Web of Science Literature from 2004 to 2024
Source: Healthcare (Basel). 2025 Oct 23;13(21):2687. doi: 10.3390/healthcare13212687 (PMC12609531; doi:10.3390/healthcare13212687)
Supplement: Supplementary file 1 [file healthcare-13-02687-s001.zip › healthcare-3766074-supplementary/Figure S2.pdf]

Top 25 References with the Strongest Citation Bursts

References	Year	Strength	Begin	End	2014 - 2024
Barnett K, 2012, LANCET, V380, P37, DOI 10.1016/S0140-6736(12)60240-2, DOI	2012	**166.09**	2014	2017	
Marengoni A, 2011, AGEING RES REV, V10, P430, DOI 10.1016/j.arr.2011.03.003, DOI	2011	**65.01**	2014	2016	
Fortin M, 2012, ANN FAM MED, V10, P142, DOI 10.1370/afm.1337, DOI	2012	**56.05**	2014	2017	
Violan C, 2014, PLOS ONE, V9, P0, DOI 10.1371/journal.pone.0102149, DOI	2014	**49.65**	2016	2019	
Nunes BP, 2016, ARCH GERONTOL GERIAT, V67, P130, DOI 10.1016/j.archger.2016.07.008, DOI	2016	**45.94**	2018	2021	
Salisbury C, 2011, BRIT J GEN PRACT, V61, P0, DOI 10.3399/bjgp11X548929, DOI	2011	**41.13**	2014	2016	
Salive ME, 2013, EPIDEMIOL REV, V35, P75, DOI 10.1093/epirev/mxs009, DOI	2013	**35.87**	2015	2018	
Wallace E, 2015, BMJ-BRIT MED J, V350, P0, DOI 10.1136/bmj.h176, DOI	2015	**33.88**	2016	2020	
Masnoon N, 2017, BMC GERIATR, V17, P0, DOI 10.1186/s12877-017-0621-2, DOI	2017	**33.79**	2019	2022	
Read JR, 2017, J AFFECT DISORDERS, V221, P36, DOI 10.1016/j.jad.2017.06.009, DOI	2017	**33.79**	2019	2022	
Garin N, 2016, J GERONTOL A-BIOL, V71, P205, DOI 10.1093/gerona/glv128, DOI	2016	**32.98**	2018	2021	
Cassell A, 2018, BRIT J GEN PRACT, V68, PE245, DOI 10.3399/bjgp18X695465, DOI	2018	**32.28**	2020	2024	
Diederichs C, 2011, J GERONTOL A-BIOL, V66, P301, DOI 10.1093/gerona/glq208, DOI	2011	**31.68**	2014	2016	
Smith SM, 2016, COCHRANE DB SYST REV, V0, P0, DOI 10.1002/14651858.CD006560.pub4, DOI	2016	**31.12**	2017	2021	
Smith SM, 2012, BMJ-BRIT MED J, V345, P0, DOI 10.1136/bmj.e5205, DOI	2012	**30.92**	2014	2017	
Prados-Torres A, 2014, J CLIN EPIDEMIOL, V67, P254, DOI 10.1016/j.jclinepi.2013.09.021, DOI	2014	**30.69**	2015	2019	
Johnston MC, 2019, EUR J PUBLIC HEALTH, V29, P182, DOI 10.1093/eurpub/cky098, DOI	2019	**29.66**	2021	2024	
Pefoyo AJK, 2015, BMC PUBLIC HEALTH, V15, P0, DOI 10.1186/s12889-015-1733-2, DOI	2015	**28.98**	2016	2020	
Bähler C, 2015, BMC HEALTH SERV RES, V15, P0, DOI 10.1186/s12913-015-0698-2, DOI	2015	**28.63**	2016	2020	
Zhao Y, 2020, LANCET GLOB HEALTH, V8, PE840, DOI 10.1016/S2214-109X(20)30127-3, DOI	2020	**28.14**	2022	2024	
Huntley AL, 2012, ANN FAM MED, V10, P134, DOI 10.1370/afm.1363, DOI	2012	**28.12**	2014	2017	
Tinetti ME, 2012, JAMA-J AM MED ASSOC, V307, P2493, DOI 10.1001/jama.2012.5265, DOI	2012	**27.72**	2014	2017	
Xu XL, 2017, AGEING RES REV, V37, P53, DOI 10.1016/j.arr.2017.05.003, DOI	2017	**27.45**	2019	2022	
Glynn LG, 2011, FAM PRACT, V28, P516, DOI 10.1093/fampra/cmr013, DOI	2011	**27.45**	2014	2016	
Ho ISS, 2021, LANCET PUBLIC HEALTH, V6, PE587, DOI 10.1016/S2468-2667(21)00107-9, DOI	2021	**25.9**	2022	2024	

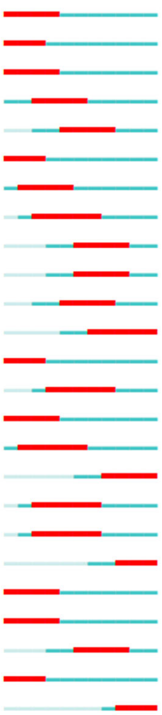

Figure S2. Prominent Publications in Multimorbidity Research Indexed by WOS
