# Supplementary material for: The Multimorbidity Knowledge Domain: A Bibliometric Analysis of Web of Science Literature from 2004 to 2024
Source: Healthcare (Basel). 2025 Oct 23;13(21):2687. doi: 10.3390/healthcare13212687 (PMC12609531; doi:10.3390/healthcare13212687)
Supplement: Supplementary file 1 [file healthcare-13-02687-s001.zip › healthcare-3766074-supplementary/Table S1.pdf]

Table S1. Keyword Frequency in Multimorbidity Research Indexed in WOS

Year	Keyword	Betweenness	N	Year	Keyword	Betweenness	N
2004	health	0.31	1171	2016	anxiety	0.25	122
2004	prevalence	0.26	1713	2016	hospital admissions	0.18	6
2004	body mass index	0.19	99	2017	mental disorders	0.06	20
2004	epidemiology	0.06	480	2018	cluster analysis	0.05	34
2005	comorbidity	0.67	738	2019	inflammation	0.06	80
2005	family practice	0.64	23	2019	home	0.04	7
2005	morbidity	0.57	249	2019	depressive symptoms	0.02	89
2005	chronic disease	0.42	541	2020	functional status	0.04	20
2005	population	0.37	698	2020	inequality	0.02	50
2006	depression	0.32	477	2020	medication adherence	0.02	44
2006	age	0.18	269	2020	pain	0.02	32
2006	health services research	0.04	10	2021	prediction	0.05	9
2006	classification	0.02	26	2021	multimorbidity patterns	0.04	32
2007	chronic diseases	0.43	448	2021	machine learning	0.04	31
2007	disability	0.31	387	2021	adolescents	0.03	8
2007	therapy	0.23	72	2021	infection	0.02	19
2007	elderly patients	0.18	204	2022	cardiometabolic multimorbidity	0.08	66
2007	community	0.18	116	2022	social determinants	0.01	9
2007	impact	0.17	728	2022	cohort study	0	40
2008	outcome	0.18	551	2023	air pollution	0.05	18
2008	coronary heart disease	0.17	55	2023	overweight	0.03	18
2008	dementia	0.16	199	2023	mobility	0.02	11
2009	adults	0.12	745	2024	life style	0	17
2009	association	0.12	678	2024	long-term care	0	11
2009	risk	0.11	1008	2024	exposure	0	10
2009	diabetes mellitus	0.11	126	2024	systematic review	0	10
2010	co morbidity	0.3	70	2024	cardiometabolic diseases	0	9
2012	cardiovascular disease	0.26	277	2024	retirement	0	9
2012	interventions	0.11	112	2024	oxidative stress	0	9
2013	meta analysis	0.11	228	2024	patient-centered care	0	9
2014	socioeconomic status	0.06	103	2024	type 2 diabetes mellitus	0	9
2015	adherence	0.11	90	2024	cognitive function	0	8
