# Supplementary material for: The Multimorbidity Knowledge Domain: A Bibliometric Analysis of Web of Science Literature from 2004 to 2024
Source: Healthcare (Basel). 2025 Oct 23;13(21):2687. doi: 10.3390/healthcare13212687 (PMC12609531; doi:10.3390/healthcare13212687)
Supplement: Supplementary file 1 [file healthcare-13-02687-s001.zip › healthcare-3766074-supplementary/Table S2.pdf]

Table S2. Highly Cited Articles in Multimorbidity Research Indexed in WOS

Year	N	Betweenness	Reference
2012	424	0.43	BARNETT K, Epidemiology of multimorbidity and implications for health care, research, and medical education: a cross-sectional study, LANCET.
2019	250	0.02	Johnston MC, Defining and measuring multimorbidity: a systematic review of systematic reviews. Eur J Public Health.
2011	175	0.37	MARENGONI A, Aging with multimorbidity: A systematic review of the literature, AGEING RES REV.
2022	159	0	Skou ST, Multimorbidity. Nat Rev Dis Primers.
2012	156	0.58	FORTIN M, A Systematic Review of Prevalence Studies on Multimorbidity: Toward a More Uniform Methodology, ANN FAM MED.
2019	141	0	Makovski TT, Multimorbidity and quality of life: Systematic literature review and meta-analysis, AGEING RES REV.
2014	135	0.75	VIOLAN C, Prevalence, determinants and patterns of multimorbidity in primary care: a systematic review of observational studies, PLOS ONE.
2019	126	0.49	Nguyen Hai, Prevalence of multimorbidity in community settings: A systematic review and meta-analysis of observational studies, J COMORB.
2018	123	0.20	Cassell A, The epidemiology of multimorbidity in primary care: A retrospective cohort study, BRIT J GEN PRACT.
2019	120	0.05	Nunes BP, Frailty and Multimorbidity: A Systematic Review and Meta-analysis, J GERONTOL A-BIOL.
2016	120	016	Nunes BP, Multimorbidity and mortality in older adults: A systematic review and meta-analysis, ARCH GERONTOL GERIAT.
