# Supplementary material for: The Multimorbidity Knowledge Domain: A Bibliometric Analysis of Web of Science Literature from 2004 to 2024
Source: Healthcare (Basel). 2025 Oct 23;13(21):2687. doi: 10.3390/healthcare13212687 (PMC12609531; doi:10.3390/healthcare13212687)
Supplement: Supplementary file 1 [file healthcare-13-02687-s001.zip › healthcare-3766074-supplementary/Table S3.pdf]

Table S3. Clustering Information of Highly Cited Publications in Multimorbidity Research Indexed by WOS.

Clusters	Size	Silhouette	mean(Year)	Top term (log-likelihood ratio, p-level)
0	29	0.959	2014	physical activity (14.36, 0.001); cardiometabolic multimorbidity (12.97, 0.001); health care utilization (12.58, 0.001); switzerland (9.9, 0.005); patient experience (8.48, 0.005)
1	25	0.98	2013	randomised controlled trial (14.78, 0.001); general practice (14.08, 0.001); primary care (13.4, 0.001); cardiometabolic multimorbidity (13.16, 0.001); qualitative research (11.64, 0.001)
2	22	0.987	2019	cardiometabolic multimorbidity (69.04, 1.0E-4); cardiometabolic disease (32.15, 1.0E-4); uk biobank (23.05, 1.0E-4); cardiometabolic diseases (20.41, 1.0E-4); cohort study (17.51, 1.0E-4)
3	19	0.947	2013	disability (17.46, 1.0E-4); function (7.27, 0.01); race (7.27, 0.01); polypharmacy (7.02, 0.01); cardiometabolic multimorbidity (6.41, 0.05)
4	19	0.967	2016	depression (13.76, 0.001); cardiometabolic multimorbidity (12.53, 0.001); healthy aging (10.73, 0.005); latent class analysis (10.19, 0.005); interaction (10.04, 0.005)
5	18	1	2014	cardiometabolic multimorbidity (9.22, 0.005); qualitative research (7.92, 0.005); heart failure (7.78, 0.01); comorbidity (6.93, 0.01); self-management (6.43, 0.05)
6	18	0.974	2019	multiple long-term conditions (20.57, 1.0E-4); frailty (11.07, 0.001); cardiometabolic multimorbidity (11.07, 0.001); systematized nomenclature of medicine (10.51, 0.005); ethnic inequalities (10.51, 0.005)
7	17	1	2021	cardiometabolic multimorbidity (23.43, 1.0E-4); dementia (17.51, 1.0E-4); comorbidity (11.92, 0.001); cerebrospinal fluid (11.75, 0.001); life style (11.75, 0.001)
8	17	0.958	2016	frailty (144.32, 1.0E-4); cardiometabolic multimorbidity (12, 0.001); community (10.98, 0.001); frailty phenotype (10.2, 0.005); geriatric assessment (10.2, 0.005)
9	16	0.978	2019	real-world evidence (11.24, 0.001); observational study (11.24, 0.001); frailty (9.18, 0.005); population study (5.94, 0.05); multimorbidity clusters (5.94, 0.05)
10	16	0.873	2013	cardiometabolic multimorbidity (12.34, 0.001); measurement (11.14, 0.001); dietary approaches to stop

				hypertension (10.09, 0.005); combinations (10.09, 0.005); dash (10.09, 0.005)
11	15	1	2016	polypharmacy (156.69, 1.0E-4); inappropriate prescribing (26.7, 1.0E-4); deprescribing (24.35, 1.0E-4); pharmacoepidemiology (19.29, 1.0E-4); pharmacotherapy (17.72, 1.0E-4)
12	15	0.929	2018	china (20.63, 1.0E-4); catastrophic health expenditure (20.53, 1.0E-4); bayesian networks (12.31, 0.001); influencing factors (12.31, 0.001); related factors (12.31, 0.001)
13	14	0.937	2019	adult (15.41, 1.0E-4); cardiometabolic multimorbidity (11.79, 0.001); geocoding (10.27, 0.005); area deprivation index (10.27, 0.005); us census (10.27, 0.005)
14	12	0.912	2015	south asia (13.97, 0.001); disability (7.89, 0.005); health (7.43, 0.01); geriatric syndrome (6.98, 0.01); vascular mortality (6.98, 0.01)
15	11	0.841	2019	cardiometabolic multimorbidity (75.45, 1.0E-4); uk biobank (18.76, 1.0E-4); prospective cohort study (12.31, 0.001); multimorbidity (12.17, 0.001); cardiometabolic disease (11.64, 0.001)
