# Supplementary figures and images for: The Multimorbidity Knowledge Domain: A Bibliometric Analysis of Web of Science Literature from 2004 to 2024
Source: Healthcare (Basel). 2025 Oct 23;13(21):2687. doi: 10.3390/healthcare13212687 (PMC12609531; doi:10.3390/healthcare13212687)

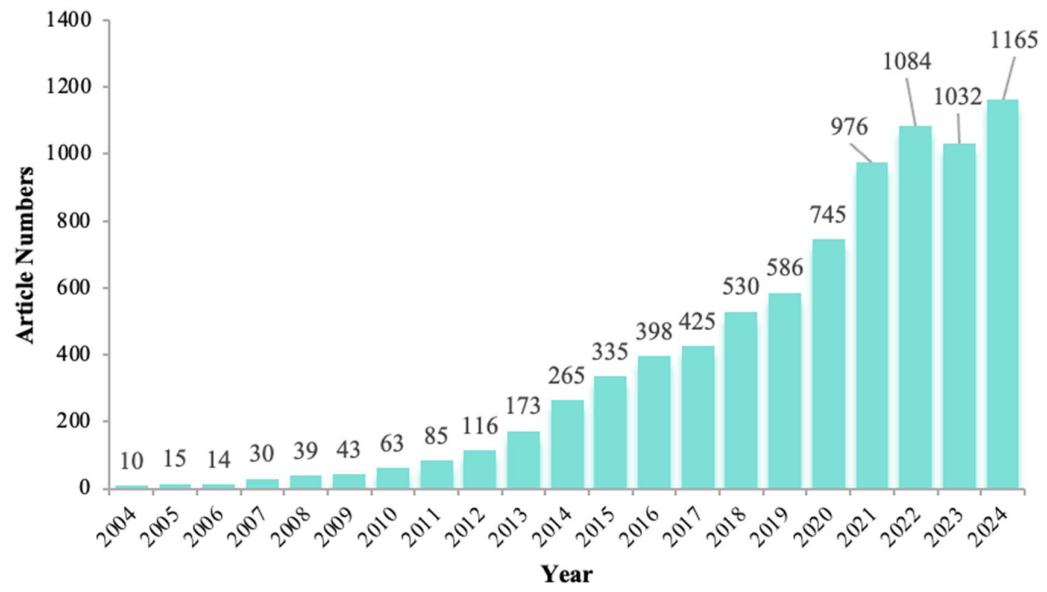

**Figure S1.** Annual Publication Volume in Multimorbidity Research

Supplement: Supplementary file 1 [file healthcare-13-02687-s001.zip › healthcare-3766074-supplementary/Figure S1.pdf]
